# Targeting CD4+ T cells through gut microbiota: therapeutic potential of traditional Chinese medicine in inflammatory bowel disease

**DOI:** 10.3389/fcimb.2025.1557331

**Published:** 2025-03-03

**Authors:** Xingyao Lu, Yichuan Xv, Weiye Hu, Boyun Sun, Hongyi Hu

**Affiliations:** ^1^ Department of Gastroenterology, Longhua Hospital, Shanghai University of Traditional Chinese Medicine, Shanghai, China; ^2^ Department of Liver Disease, Shanghai Yueyang Integrated Traditional Chinese Medicine and Western Medicine Hospital, Shanghai University of Traditional Chinese Medicine, Shanghai, China

**Keywords:** gut microbiota, CD4+ T cell, traditional Chinese medicine, inflammation bowel disease, metabolites

## Abstract

Inflammatory Bowel Disease (IBD) is an autoimmune disease characterized by chronic relapsing inflammation of the intestinal tract. Gut microbiota (GM) and CD4^+^T cells are important in the development of IBD. A lot of studies have shown that GM and their metabolites like short-chain fatty acids, bile acids and tryptophan can be involved in the differentiation of CD4^+^T cells through various mechanisms, which in turn regulate the immune homeostasis of the IBD patients. Therefore, regulating CD4^+^T cells through GM may be a potential therapeutic direction for the treatment of IBD. Many studies have shown that Traditional Chinese Medicine (TCM) formulas and some herbal extracts can affect CD4^+^T cell differentiation by regulating GM and its metabolites. In this review, we mainly focus on the role of GM and their metabolites in regulating the differentiation of CD4^+^T cells and their correlation with IBD. We also summarize the current research progress on the regulation of this process by TCM.

## 1 Introduction

Inflammatory Bowel Disease (IBD) is a chronic, immune-mediated gastrointestinal disorder characterized by relapsing episodes, primarily encompassing ulcerative colitis (UC) and Crohn’s disease (CD). These conditions share similar gastrointestinal manifestations including abdominal pain, diarrhea, and bloody stools (Torres et al., 2017; Chang, 2020; Gros and Kaplan, 2023). UC, characterized by inflammation of the mucosal and submucosal layers, starts primarily in the rectum and can progress to the entire colon. The clinical manifestations of CD are slightly different from those of UC. CD is characterized by transmural inflammation that can involve the entire digestive tract.

T cells, primarily including CD8^+^ and CD4^+^T cells, play a crucial role in the pathogenesis of IBD. Previous studies have found that cytotoxic CD8^+^ T cells (Tc1) and tissue resident memory CD8^+^ T cells enriched in inflamed colon tissues, potentially contributing to the pathogenesis of IBD. CD4^+^T cells, key cells in adaptive immune responses, have emerged as crucial targets in the treatment of IBD (Selby et al., 1984; Müller et al., 1998; Basso et al., 2018). Biologics targeting CD4^+^T cell-related inflammatory cytokines can effectively inhibit immune inflammation in IBD, bringing about a breakthrough revolution in clinical practice (D’Haens and van Deventer, 2021). However, quite a part of patients still faces the dilemma of primary or secondary loss of response to these targeted drugs. Therefore, the upstream regulatory factors of CD4^+^T-related immune response deserve further investigation.

Gut microbiota (GM), mainly composed of bacteria, archaea, fungi, and viruses, is one of the most complex microbial ecosystems in the human body. GM is an essential component of immune homeostasis (Fang et al., 2025). Substantial evidence indicates that the composition of GM and their metabolites are closely associated with immune dysregulation in IBD patients (Frank et al., 2007; Morgan et al., 2012). Evidence from animal models suggests that IBD is a multifactorial condition driven by immune dysregulation and gut dysbiosis. GM is the culprit in initiating colon inflammation, disrupting the mucosal barrier and recruiting pro-inflammatory cells to infiltrate the mucosa in the early stage. Recent researches have demonstrated an interaction between GM and CD4^+^T cells, especially in autoimmune diseases such as IBD. Dysbiosis of GM promotes the inflammation mediated by CD4^+^T cells by creating an inflammatory microenvironment and disrupting metabolic balance.

Traditional Chinese Medicine (TCM) has demonstrated good clinical efficacy in the treatment of IBD, and its mechanisms may be related to the regulation of the GM, inhibition of the inflammatory response, and repair of the damaged intestinal barrier (Shen et al., 2021; Xiaoling et al., 2024). Studies have shown that TCM administration can increase the abundance of probiotics and reduce the levels of inflammatory factors in the colon mucosa and serum (Hu et al., 2024).Further studies have shown that TCM formula or some herbal extracts can directly modulate the differentiation of CD4^+^T cells or indirectly regulate this process through their involvement in the metabolism of short-chain fatty acids (SCFAs), bile acids (BAs), and tryptophan (Trp) (Zhang et al., 2025; Wang et al., 2021). In this review, we summarize the roles of CD4^+^T cells and the GM in IBD, highlighting the regulatory role of GM and its metabolites in CD4^+^T cell differentiation. Meanwhile, we also summarize and discusses the potential of TCM in modulating the microbiota-immune axis.

## 2 GM and IBD

### 2.1 Gut dysbiosis in IBD

Studies based on experimental colitis suggested the initiating contribution of GM in colitis development. Early research has found that resident enteric baicalin teria are necessary for the development of spontaneous colitis and immune activation in interleukin-10 (IL-10)-deficient mice (Sellon et al., 1998). A recent study showed that microbial alterations are associated with early intestinal barrier dysfunction and act as an initial pathophysiological event in animal models of IBD, preceding histological inflammation (López-Cauce et al., 2022). Further studies found transplanting the GM of IBD model rats into the healthy mice can elicit a significant intestinal inflammation, confirming the causative role of gut dysbiosis in IBD (Schaubeck et al., 2016). Correspondingly, antibiotic treatment can prevent or mitigate the development of IL-10-deficient colitis in animal models (Tamagawa et al., 2007).

In most healthy individuals, intestinal bacteria are mainly composed of Firmicutes, Bacteroidetes, Proteobacteria, and Actinobacteria, with Firmicutes and Bacteroidetes comprising about 90% of the bacteria (Eckburg et al., 2005; Ley et al., 2008). Clinical studies have demonstrated significant differences in GM between IBD patients and healthy population. UC and CD patients consistently exhibit a decrease in the relative abundance of Firmicutes and Bacteroidetes, accompanied by an increase in Proteobacteria and Actinobacteria at the phylum level (Frank et al., 2007; Morgan et al., 2012). Furthermore, the abundances of Roseburia and Phascolarctobacterium are significantly reduced in both UC and CD at the genus level (Morgan et al., 2012; Lewis et al., 2015; Yilmaz et al., 2019). An increased abundance of adherent-invasive *Escherichia coli* (AIEC) has been observed in the ileum and colon of IBD patients, especially in CD (Chervy et al., 2020). Although UC and CD share certain inflammatory pathways and microbial features, there are still differences in specific microbial alterations. A study using 16S rRNA to analyze 2,045 fecal samples from both non-IBD and IBD patients across four countries and observed greater gut dysbiosis in CD, including a lower microbial diversity, and higher variation in microbiome composition in the Spanish cohort. Researchers also found that GM can be used to differentiate CD and UC. Faecalibacterium, Peptostreptococcaceae, Anaerostipes, Methanobrevibacter, Collinsella, and Christensenellaceae were more abundant in UC, whereas Fusobacterium and Escherichia were enriched in CD (Pascal et al., 2017). An increased abundance of AIEC has been observed in the ileum and colon of IBD patients, especially in CD. AIEC is capable of interacting with intestinal epithelial cells (IECs) and immune cells, leading to disruption of the intestinal barrier and promotion of pro-inflammatory cytokine (Chervy et al., 2020). The specific change of gut bacteria in UC and CD patients are summarized in detail in **Table 1**.

**Table 1:** Bacteria dysbiosis in IBD patients.

Type	Method	Level	Gut dysbiosis	Reference
CD	16S rRNA+ metagenomics sequencing	Phylum	Bacteroidetes↑	(Gevers et al., 2014)
		Order	Erysipelotrichales, Bacteroidales, Clostridiales ↓	
		Family	Enterobacteriaceae, Pasteurellacaea, Veillonellaceae, Fusobacteriaceae↑	
CD	16S rRNA	Species	*Clostridium cluster* XIVa*, Dialister invisus, F. prausnitzii, B. adolescentis*↓ *Ruminococcus gnavus* ↑	(Joossens et al., 2011)
CD	metagenomic sequencing	Genus	*Prevotella, Eubacterium, Odoribacter, Akkermansia, Roseburia, Parabacteroides, Alistipes, Coprococcus, Dorea, Ruminococcus* ↓ *Escherichia, Klebsiella, Enterococcus, Veillonella* ↑	(Lewis et al., 2015)
CD	16S rRNA	Genus	*Bacteroides, Eubacterium, Faecalibacterium, Ruminococcus* ↓ *Actinomyces, Bifidobacterium* ↑	(Takahashi et al., 2016)
		Species	*Blautia faecis, Roseburia inulinivorans, Ruminococcus torques, Clostridium lavalense, Bacteroides uniformis, F. prausnitzii* ↓	
CD	RT-PCR	Species	*F. prausnitzii* ↓	(Fujimoto et al., 2013)
CD	16S rRNA	Phylum	Proteobacteria, Bacteroidetes*↑*	(Gophna et al., 2006)
CD	16S rRNA	Genus	*Lachnospira*, *Blautia, Dorea, Coprococcus*, *Roseburia*, *Oscillospira*, *Bilophila* ↓	(Yilmaz et al., 2019)
UC	16S rRNA	Phylum	Firmicutes, Bacteroidetes ↓ Proteobacteria ↑	(Walujkar et al., 2014)
UC	RT-PCR	Species	*Roseburia hominis*, *F. prausnitzii*↓	(Machiels et al., 2014)
UC	16S rRNA	Genus	*Roseburia* ↓	(Bajer et al., 2017)
		Species	*Akkermansia muciniphila*, *Butyricicoccus pullicaecorum*, *Clostridium colinum*↓	
UC+CD	16S rRNA+ metagenomic sequencing	Genus	In CD and UC: *Roseburia*, *Phascolarctobacterium* ↓ *Clostridium* ↑	(Morgan et al., 2012)
UC+CD	16S rRNA	Family	In CD: Peptostreptococcaceae, Christensenellaceae↓ In UC: Peptostreptococcaceae ↑	(Pascal et al., 2017)
		Genus	abundant UC and absent or almost absent in CD: *Faecalibacterium*, *Anaerostipes*, *Methanobrevibacter, Collinsella* abundant CD and absent or almost absent in UC: *Fusobacterium*, *Escherichia*	
UC+CD	metagenomic sequencing	Family	Neisseriaceae, Fusobacteria, Enterobacteriaceae, Pasteurellaceae *↑*	(Ryvchin et al., 2021)
UC+CD	metagenomic sequencing	Species	*Roseburia inulinivorans*, *Burkholderiales* sp*ecies↑*	(Ananthakrishnan et al., 2017)
UC+CD	16S rRNA	Phylum	In CD: Firmicutes, Bacteroidetes ↓ Proteobacteria ↑	(Nishino et al., 2018)
		Genus	In CD: *Escherichia, Ruminococcus, Cetobacterium, Actinobacillus Enterococcus* ↑ *Faecalibacterium, Coprococcus, Prevotella*, *Roseburia* ↓	
IBD	qPCR	Species	*F. prausnitzii↓* *Escherichia coli↑*	(Lopez-Siles et al., 2014)

↑, upregulated; ↓, dwonregulated.

Most researches about gut dysbiosis have primarily focused on bacteria, yet fungi, viruses, and archaea also play significant roles in the pathogenesis of IBD (Sokol et al., 2017; Maronek et al., 2020; Houshyar et al., 2021). Studies have demonstrated that in IBD patients, the ratio of Basidiomycota to Ascomycota, as well as the abundance of *Candida albicans*, are significantly increased compared to healthy individuals. In contrast, the abundance of *Saccharomyces cerevisiae* is markedly reduced (Chehoud et al., 2015; Sokol et al., 2017). Anti-Saccharomyces cerevisiae antibody is also a biomarker for CD (Giaffer et al., 1992; Quinton et al., 1998). Notably, *Saccharomyces cerevisiae* has been shown to alleviate intestinal inflammation by inhibiting the colonization of the intestinal mucosa by AIEC, suggesting the close interaction between fungi and bacteria (Liguori et al., 2016). Additionally, fungal dysbiosis is more pronounced in CD patients than in those with UC (Chehoud et al., 2015; Sokol et al., 2017). Bacteriophages is the most common virus of the GM (Breitbart et al., 2003). A significant expansion of *Caudovirales* bacteriophages is the major virome change in IBD patients, which may be associated with decreased bacterial diversity (Norman et al., 2015; Zuo et al., 2019). As for archaea, Methanogens, the predominant archaeal group, are more abundant in the ileum and colon of IBD patients, especially UC patients (Massimino et al., 2021).

### 2.2 Microbial metabolites and IBD

#### 2.2.1 SCFAs

GM ferments non-digestible polysaccharides such as dietary fiber into SCFAs, such as acetate, propionate, and butyrate (Rowland et al., 2018). SCFAs are the major source of energy for the colonic epithelium, playing a role in maintaining the integrity of the intestinal barrier. Studies found that the level of SCFAs in the feces of IBD patients were significantly lower than those of normal individuals, which is more pronounced during the active phase of the disease (Kumari et al., 2013; Lloyd-Price et al., 2019). In IBD patients, SCFAs-producing bacterial species such as *Roseburia inulinivorans*, *Roseburia hominis, Ruminococcus torques*, *Clostridium lavalense*, *Bacteroides uniformis*, *Phascolarctobacterium* and *Faecalibacterium prausnitzii* (*F. prausnitzii*) were significantly reduced (Machiels et al., 2014; Takahashi et al., 2016). Among all SCFAs, the function of butyrate in IBD has been relatively well studied (Haneishi et al., 2023). Butyrate can maintain intestinal mucosal integrity by strengthening connections between IECs and promoting IECs regeneration. Butyrate also regulates innate and adaptive immune responses by modulating macrophage (Chang et al., 2014) and CD4^+^T cell differentiation (Arpaia et al., 2013) and inhibiting the pro-inflammatory ability of neutrophils and formation of the neutrophil extracellular traps (NETs) (Li et al., 2021a).

#### 2.2.2 BAs

BAs is important in lipid absorption and immune homeostasis (Hang et al., 2019; Song et al., 2020). There is disruption in BAs metabolism of IBD patients, characterized by an increased level of primary bile acids (PBAs) (e.g., cholic acid (CA) and chenodeoxycholic acid (CDCA)) and a decreased level of secondary bile acids (SBAs) (e.g., deoxycholic acid (DCA) and lithocholic acid (LCA)) (Franzosa et al., 2019). CD patients with lower serum levels of DCA tend to less respond to anti-TNF therapy (Ding et al., 2020). GM deeply involved in the production of SBAs and BA-derivatives through deconjugation, desulphation, dehydrogenation, dehydroxylation, and epimerization (Wahlström et al., 2016). Bile salt hydrolase (BSH) enzymes are able to deconjugate glycine or taurinebound PBAs. The abundance of specific bacteria that contain BSH like *Clostridium*, *Bifidobacterium*, *Bacteroides* and *Lactobacillus* have been found to be decreased in IBD. Some species of the genus *Clostridium* including *Clostridium scindens*, *Clostridium hiranonis*, and *Clostridium hylemonae* can convert PBAs to SBAs by dihydroxylation (Guzior and Quinn, 2021). BAs and BA-derivatives contribute to maintaining intestinal barrier, reducing secretion of inflammation-related molecules, and regulating the differentiation of immune cells via binding to certain receptors. Additionally, the decreased level of SBAs in IBD may increase the risk of *Clostridium difficile* in IBD patients (Allegretti et al., 2016).

#### 2.2.3 Trp

Trp is an essential amino acid in humans and is mainly metabolized through the kynurenine (Kyn) pathway. Trp is degraded into Kyn through indoleamine 2,3-dioxygenase (IDO) and tryptophan 2,3-dioxygenase, and further metabolized into active molecules such as quinolinic acid and picolinic acid through a series of enzymes. Besides, approximately 5% of Trp is metabolized through the GM and transformed into indole and its derivatives (Gao et al., 2018; Xue et al., 2023). In IBD patients, microbiota metabolic pathway for Trp is inhibited, whereas the Kyn pathway is relatively increased. Compared to the normal population, IBD patients, particularly those with CD, have elevated Kyn/Trp ratios and quinolinic acid levels, along with lower levels of indole and related metabolites (Gupta et al., 2012; Lee et al., 2014; Nikolaus et al., 2017). *Bacteroides*, *Bifidobacterium*, *Clostridium*, *Akkermansia*, and *Lactobacillus* can metabolize Trp into indole metabolites (Agus et al., 2018; Roager and Licht, 2018). The abundance of these bacteria is significantly reduced in IBD patients, suggesting the alteration may be shaped by gut dysbiosis. Indole can enhance the intestinal epithelial barrier by strengthening the tight junctions between IECs. Indole and related metabolites are important ligands for aryl hydrocarbon receptors (AHR). AHR is important for modulating the immune homeostasis of CD4^+^T cells, which will be discussed in the later section.

## 3 CD4^+^ T cell and IBD

Under specific exogenous stimulation, naïve CD4^+^T cells can differentiate into different subsets including T helper 1 (Th1), Th2, Th9, Th17, Th22, and regulatory T (Treg) cells (Gomez-Bris et al., 2023). They secrete distinct cytokines to interact with IECs or other immune cells, thereby exerting pro-inflammatory or anti-inflammatory effects. Notably, although UC and CD share some immunologic features mediated by CD4^+^ T cells, there are some differences in immune microenvironments. UC exhibits more prominent neutrophilic inflammation activated by Th17, such as the formation of NETs. CD is associated with more pronounced Th1 and Th17 immune responses compared to UC (Mitsialis et al., 2020).

### 3.1 Th1

The differentiation of Th1 cells depends on the activation of the STAT4 signaling pathway, which is induced by IL-12 and IL-18 secreted by antigen presenting cells (APCs). STAT4 phosphorylation induces the production of interferon-γ (IFN-γ) and T-bet, which are specific factors for the Th1 cell program. Th1 cells play an essential role in maintaining intestinal homeostasis (Gomez-Bris et al., 2023). However, the excessive Th1 cell immune responses were thought to be involved in the onset of mucosal inflammation of IBD (Gwela et al., 2017; Zorzi et al., 2013). Epithelium-infiltrated IFN-γ can directly induce the chemotaxis of macrophages and neutrophils by prompting IECs to express adhesion molecules. Tumor necrosis factor-α (TNF-α) in the epithelium activates the apoptosis pathway, induces IECs apoptosis, and further exacerbates mucosal inflammation (Alfen et al., 2018; Li et al., 2019). IFN-γ can also drive CD8^+^cells to differentiate into Tc1 cells (Mittrücker et al., 2014) and produce more pro-inflammatory molecules including IFN-γ, TNF-α, granzyme B and perforin (St Paul and Ohashi, 2020), which play a role in the initiation and development of colitis (Nancey et al., 2006; Westendorf et al., 2006). Increased numbers of Th1 cells and Th1 cell-related cytokines, such as IFN-γ, IL-2, and TNF-α, can be detected in the intestinal mucosal tissue and peripheral blood of patients with UC and CD. In IBD mouse model, a lack of IFN-γ in CD4 T-cells prevents the development of Dextran Sulfate Sodium (DSS)-induced colitis (Zimmermann et al., 2016).

### 3.2 Th2

Th2 cells differentiation is believed to be induced by activation of the STAT-6 pathway and transcription of GATA binding protein 3 (GATA-3) under IL-4 stimulation (Walker and McKenzie, 2018). Increased Th2 cells cytokines IL-4, IL-5, and IL-13 can be observed in UC and CD (Nemeth et al., 2017; Walker and McKenzie, 2018). IL-4 can also drive the differentiation of Tc2 cells, stimulating them to secrete more IL-4, IL-5, and IL-13 (Mittrücker et al., 2014). Although the immune inflammation in UC is generally believed to be Th2 cells-mediated, the use of anti-IL-13 monoclonal antibodies troleumab and onlucumab did not produced clinical benefits. Interestingly, current clues suggest that Th2 cells seem to be involved in the fibrosis in the later stages of inflammation. In a trinitrobenzene sulfonic acid (TNBS)-induced colitis model, IL-13 production begins on day 28 and peaks on day 49 and is related to increased collagen (fibrosis) production (Fichtner-Feigl et al., 2008). A clinical study found that in the early stage of UC, the expression of Th1-related genes increased, while in the late stage of the disease (10 years), the expression of Th2 cells -related genes like IL4R, GFI1, IL1RL1, PPARG, and IL5 increased (Mavroudis et al., 2019). Thus, the role of Th2 cells in IBD still needs to be further explored.

### 3.3 Th17

Th17 cells are characteristically express Retinoic acid receptor-related orphan receptor gamma t (RORγt) and secrete IL-17, whose differentiation is usually initiated by transforming growth factor-β (TGF-β) and IL-6. They activate STAT3 and SMAD pathways, induce high expression of RORγt, then stimulate the transcription of IL-17 (Jiang et al., 2023). In addition, IL-23 is very important for Th17 cells. Although it cannot induce the differentiation of Th17 cells, it can change the metabolic state of Th17 cells, which is essential for maintaining its differentiation and inducing its pathogenicity (Wu and Wan, 2020). Th17 cells play a key role in the pathogenesis of IBD. Excessive IL-17 and IL-21 in the mucosa can stimulate myofibroblasts to secrete matrix metalloproteinases, leading to epithelial cell damage (Monteleone et al., 2012). In addition, IL-17 can synergize with TNF-α to activate NF-κB, ERK1/2, and p38 signaling pathways, induce enteric neuroendocrine cells and goblet cells to secrete IL-17C, and promote IECs to secrete CCL20, a potent chemokine for Th17 (Song and Qian, 2013). IL-17A can promote IECs to secrete IL-8, which can stimulate neutrophils and promote NETs formation. At the same time, many studies have found that excessive immune activation of Th1 cells during the development of UC is often accompanied by enhanced immune response of Th17 cells, proving that Th1 cells and Th17 cells have a synergistic effect.

### 3.4 Th9

Th9 cells are a subset of CD4^+^T cells characterized by their secretion of IL-9. Its differentiation programming is regulated by the transcription factor PU.1 and induced by the combination of IL-4 and TGF-β (Neurath and Finotto, 2016). Expansion of PU.1^+^ lamina propria CD4^+^ T-cells is observed in UC, but not in CD (Nalleweg et al., 2015). The level of IL-9 mRNA expression in colon tissue from UC patients positively correlates with the endoscopic and histological disease score (Nalleweg et al., 2015). Experimental colitis studies explored the pathogenic mechanism of CD4^+^T cell-derived IL-9 in IBD. IL-9 can induce mast cell activation, which secretes trypsin and chymotrypsin, increasing intestinal permeability during IBD (Mukai et al., 2018). In TNBS-induced colitis, IL-9 deficiency led to milder intestinal inflammation, accompanied by reduced Claudin1 level and increased occludin, Claudin4 and Claudin7 level (Gerlach et al., 2015). In the oxazolidinone-colitis model, wild-type mice showed a compromised intestinal barrier compared to IL-9-deficient mice, which led to an increased bacterial entry into the mucosa (Gerlach et al., 2014). This suggests that IL-9 may contribute to IBD pathogenesis by impairing the intestinal barrier, thereby facilitating bacterial translocation into the mucosa and triggering a pro-inflammatory response.

### 3.5 Treg

Treg cells can be divided into natural regulatory T cells (nTreg) and inducible regulatory T cells (iTreg). nTreg cells are primarily generated in the thymus, where they undergo differentiation in response to self-antigens recognized via their T cell receptors to produce immune tolerance. While iTreg cells are usually formed in peripheral tissues, especially under the stimulation of specific environmental cues, notably cytokines such as TGF-β and IL-2 (Bilate and Lafaille, 2012). Treg cells, which express forkhead box protein P3 (Foxp3), are primarily secrete anti-inflammatory cytokines such as TGF-β, IL-10, and IL-35 (Zhang et al., 2023). Treg cells play a key role in immune regulation by suppressing various immune responses and inflammation (Omenetti and Pizarro, 2015). Evidence from animal models showed that depletion of Treg cells in the intestines of C57BL/6 mice exacerbate DSS-induced colitis by triggering aberrant innate immune responses, and transfer of Treg cells into colitis mice can lead to resolution of colonic inflammation (Pedros et al., 2016). Treg cells maintain intestinal epithelial stability by regulating the proliferation of epithelial stem cells and the sensitivity of IECs to inflammatory factors. A study using colon organoid have shown that Treg cells produce IL-10 to promote stem cell renewal (Biton et al., 2018). In addition, Treg cells-derived IL-10 limits mucosal inflammation by reducing the sensitivity of IECs to inflammatory cytokines and to T cell-mediated apoptosis (Bharhani et al., 2006).

### 3.6 Th22

Th22 cells are a subset of CD4^+^T cells that secrete IL-22 but do not produce IL-17 and IFN-γ. The initiation of Th22 cell differentiation is mediated by the combined action of cytokines including IL-6 and TNF-α (Dudakov et al., 2017). Current studies have shown that IL-22 play an important role in maintaining intestinal epithelial homeostasis by binding to the receptor IL-22R, the expression of which is mainly limited to epithelial cells (Lindemans et al., 2015). In a study of colon organoids, it was found that recombinant IL-22 directly targeted intestinal stem cells, promoting intestinal stem cell expansion in a STAT3-dependent manner (Lindemans et al., 2015). In a study based on colitis mouse model, IL-22 expression was found to be significantly reduced. IL-22 gene delivery by a local gene delivery system specifically enhanced STAT3 activation in colonic epithelial cells, inducing the expression of mucus-related molecules and the restoration of mucus-producing goblet cells (Sugimoto et al., 2008). In addition, IL-22 have been observed to promote the expression of antimicrobial peptides-related genes such as REG1A, REG1B, and DMBT1 to enhance the intestinal mucosal barrier. However, elevated IL-22 levels can be detrimental. IL-22 regulates proinflammatory pathways involved in immune cell chemotaxis, particularly those involving CXCR2+ neutrophils (Xv et al., 2024b). In UC patients, enrichment of IL-22 pathway genes in colon biopsies correlated with colonic neutrophil infiltration and was enriched in patients who did not respond to ustekinumab treatment (Pavlidis et al., 2022).

## 4 GM and CD4^+^ T cells differentiation

The dynamics of naïve CD4^+^T cells underscore the plasticity within the immune system, highlighting the adaptability of CD4^+^T cells in response to environmental cues. Under homeostatic conditions, pro-inflammatory and anti-inflammatory CD4^+^T cells exist in a dynamic equilibrium, where they work together to maintain the intestinal microenvironment and prevent pathogen invasion. Since GM is deeply involve in the regulation of intestinal function, the relationship between GM and CD4^+^T cells is central to maintaining mucosal barrier integrity and normal immune response. Disruptions in this communication are linked to many gastrointestinal diseases, including IBD (Xv et al., 2024a). In this section, we will discuss in depth how GM and their metabolites influence immune responses.

### 4.1 Regulating through inflammatory microenvironment

**Figure 1:**
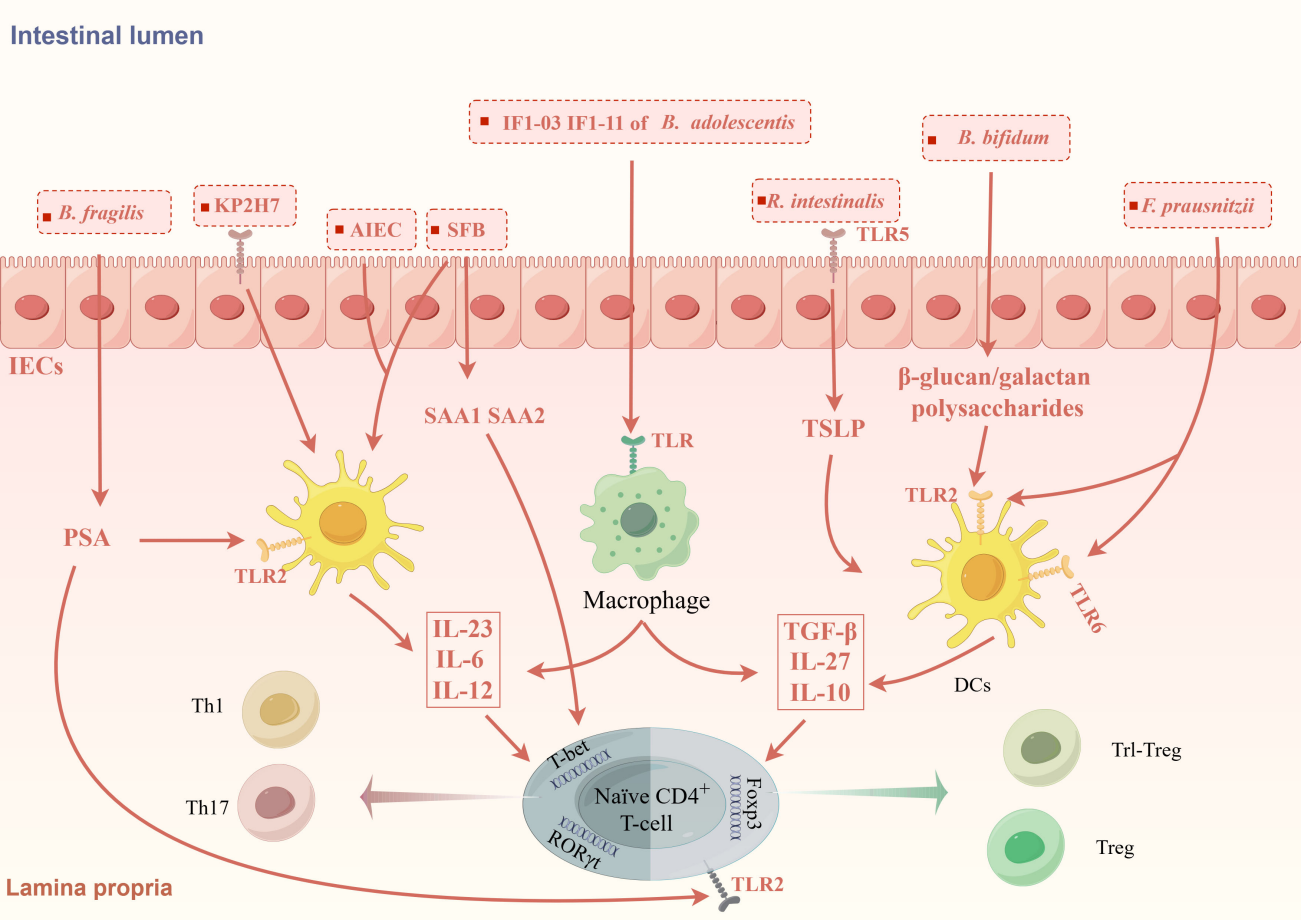
GM modulates the differentiation of CD4^+^T cells through the inflammatory microenvironment. GM promotes the formation of different inflammatory microenvironments induces CD4^+^T cells differentiation. KP2H7, AIEC and SFB can promote the production of IL-23, IL-6, IL-12 via TLRs on IECs and DCs, thereby promoting the differentiation of Th1 and Th17 cells. SFB also promotes the secretion of SAA via IECs to promote the differentiation of Th17 cells. *Bifidobacterium bifidum* (*B. bifidum*), *Roseburia intestinalis* (*R. intestinalis*) and *F prausnitzii* can promote the expression of IL-10, TGF-β, IL-27, CD39, and IDO-1 by acting on TLR receptors on DCs and IECs, which in turn promotes the differentiation of Foxp3^+^Treg cells and Foxp3^-^Tr1 cells. Different strains of *B. adolescentis* regulate Th17/Treg homeostasis by different inflammatory microenvironments. Polysaccharide A produced by *B. fragilis* can mediate the differentiation of CD4^+^T cells into different subtypes.

Studies have proven that GM can regulate the differentiation of CD4^+^ T cells by acting on APCs and IECs to promote certain cytokines (**Figure 1**). A recent study transplanted nine types of bacteria, including *Escherichia coli* and *Klebsiella pneumoniae*, isolated from the small intestine of CD patients into germ-free mice and found that the number of Th1 and Th17 cells in the intestine of mice increased. Specifically, *Escherichia coli* 35A1 induced the production of Th1 cells in a strain-specific manner, but the exact mechanism still needs exploration (Nagayama et al., 2020). *Klebsiella pneumoniae* is a kind of oral bacterium usually ectopic presence in the intestine of IBD patients, in which *Klebsiella pneumoniae* 2H7 may create a microenvironment that promotes Th1 cells differentiation through Toll-like receptors (TLRs) on IECs (Atarashi et al., 2017). GM also deeply involved in Th17/Treg differentiation. SFB is the first microbiota taxa found to be associated with Th17 cells differentiation (Ivanov and Littman, 2010). SFB can promote the production of SAA1 and SAA2 by IECs and IL-23 by dendritic cells (DCs) (Goto et al., 2014). SAA may promote Th17 differentiation by activating RORγt in a TGF-β-independent mechanism (Lee et al., 2020). The flagellin of SFB may play an important role in this process (Wang et al., 2019).IL-23 is important in maintaining the differentiation and allow Th17 cells acquire pathogenicity. At the same time, IL-23 can amplify this inflammatory cycle by promoting SAA secretion via IL-22 secreted by ILCs (Sano et al., 2015). Pathogenic Th17 (pTh17) cells are a special type of Th17 cells characterized by the expression of IL-17A, IL-22, IFN-γ, GM-CSF and are closely associated with intestinal inflammation in IBD. AIEC promotes pTh17 differentiation promoting the secretion of IL-23 from DCs. Further studies revealed that *rfaP* (LPS-core heptose kinase) and *ybaT* (inner membrane transport protein) in AIEC were responsible for promoting the transition of non-pathogenic Th17 cells to pTh17 cells (Paroni et al., 2023; Leccese et al., 2024). These GM-induced cytokines comprise an inflammatory microenvironment that can promote and maintain Th17 differentiation and pathogenicity.

Some GM taxa, on the other hand, are associated with the production of Treg cells (**Figure 1**). TLRs are expressed in many different types of immune cells and epithelial cells and can recognize microbe-associated molecular patterns, which usually are specific structural components of bacteria. The β-glucan/galactan polysaccharides derived from the cell wall of *Bifidobacterium bifidum* can promote Treg cells induction by acting on TLR2 expressed on intestinal DCs (Verma et al., 2018). *Roseburia intestinalis* can promote thymic stromal lymphopoietin (TSLP) expression via TLR5 on IECs. TSLP can promote Treg cells differentiation by inducing DCs secreting IL-10 and TGF-β (Shen et al., 2022). In addition to inducing the differentiation of classic Foxp3^+^Tregs, GM are also involved in the differentiation of some special types of Treg cells like Tr1-like Tregs (Tr1 cells). Tr1 cells is characterized by IL-10 secretion and negative Foxp3 expression, is significant decreased in IBD patients. *F. prausnitzii* can activates TLR2/6 on DCs, which further activates the downstream JNK pathway to produce cytokines including IL-10, IL-27, CD39, and IDO-1 to promote Tr1-like Treg differentiation (Alameddine et al., 2019).

### 4.2 Regulating through microbial metabolites

#### 4.2.1 SCFAs

**Figure 2:**
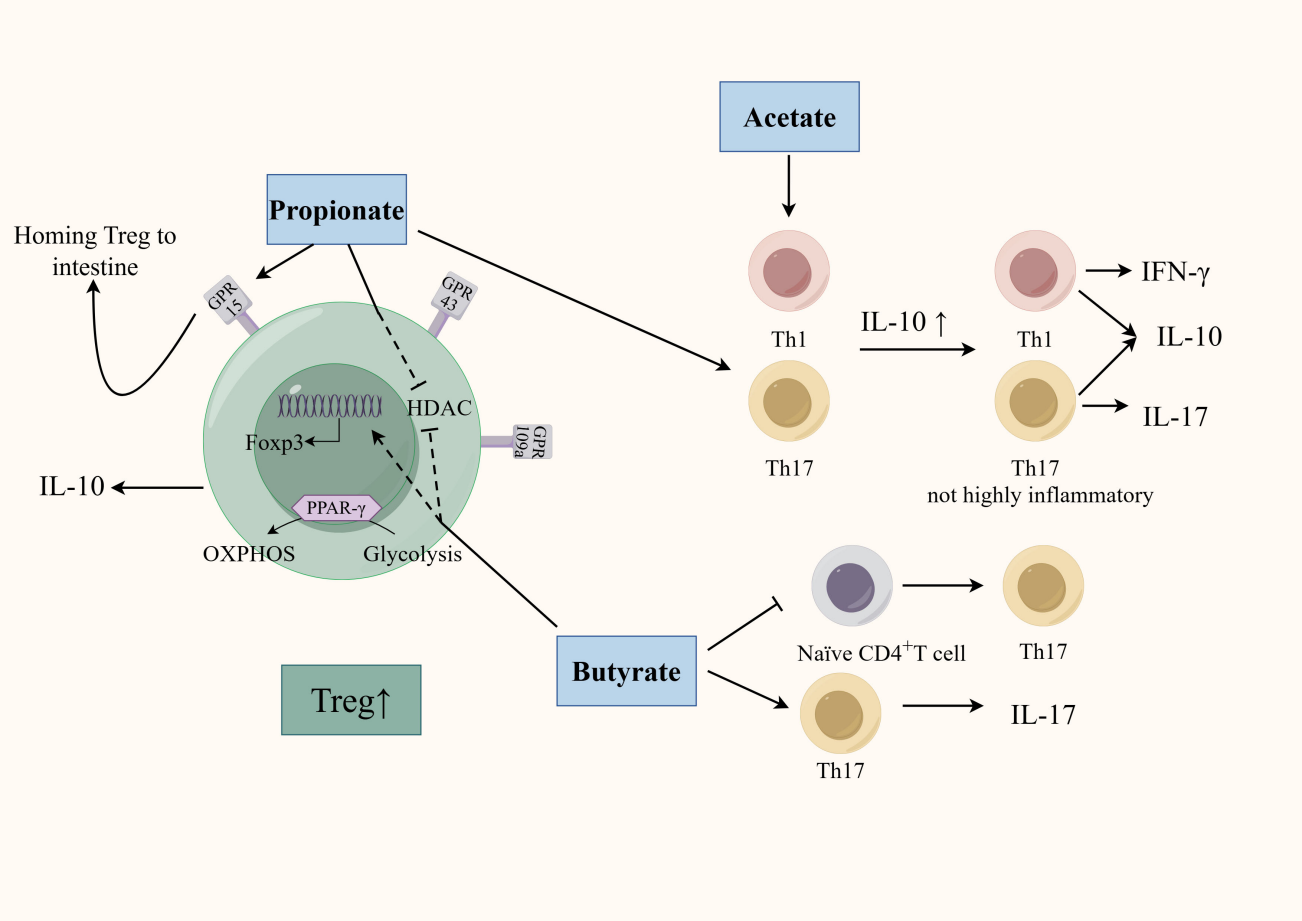
Regulation of SCFAs on the differentiation of CD4^+^T cells. SCFAs (mainly including acetate, propionate, and butyrate) can affect CD4^+^T cell differentiation through multiple mechanisms. SCFAs regulate HDAC activity and promote Treg cells differentiation in a GPR-dependent manner. Propionate activates GPR15 to promote Treg homing. Butyrate promotes Treg cell differentiation by activating PPARγ, which shifts energy metabolism from glycolysis to OXPHOS. Acetate- and propionate-induced effector T cell differentiation is less inflammatory by promoting IL-10 secretion. The regulatory role of butyrate also correlates with the differentiated status of CD4^+^T cells.

GM is deeply involved in the regulation of CD4^+^T cell differentiation through SCFAs (**Figure 2**). Earlier studies found clusters IV, XIVa, and XVIII of Clostridia could promote Treg cells differentiation through SCFAs (Atarashi et al., 2011) and *F. prausnitzii* can regulate Th17/Treg differentiation through butyrate (Zhou et al., 2018). SCFAs, especially propionate and butyrate, can inhibit the differentiation of Th1 cells, Th17 cells, Th22 cells and promote the differentiation of Treg cells through various mechanisms (Arpaia et al., 2013; Kibbie et al., 2021). Firstly, SCFAs accelerates the differentiation of colonic Treg cells in conjunction with an increase in histone H3 acetylation in the promoter and conserved non-coding sequence regions of the Foxp3 locus (Furusawa et al., 2013). Specifically, propionate regulates the activity of histone deacetylase (HDAC) through G protein-coupled receptor (GPCR) signaling to regulate Foxp3 expression. SCFAs can specifically affect HDAC6 and HDAC9 in a GPR43-dependent manner, promoting the differentiation of Foxp3^+^Treg cells in the intestine of germ-free mice (Smith et al., 2013). Using SCFAs to treat T cell transfer model mice can significantly alleviate colon inflammation and increase the proportion of Treg cells in the colon’s lamina propria. Interestingly, although TGF-β is also a characteristic cytokine secreted by Treg cells, Treg cells induced by SCFAs only specifically and highly express IL-10 without affecting the secretion of TGF-β (Smith et al., 2013). Propionate can also active GPR15, which induces Treg cells homing to the colon (Kim et al., 2013). Butyrate can inhibit HDAC1 and may participate in Treg cells differentiation by activating GPR109a (Singh et al., 2014; Zhou et al., 2018). Additionally, SCFAs can affect Treg cells differentiation though energy metabolism. SCFAs can directly affect the ATP/ADP levels through their integration in the Krebs cycle as Acetyl-CoA. This leads to the activation of the mammalian target of rapamycin, a critical kinase involved in T-cell differentiation (Luo et al., 2017). Th17 cells depend more on glycolysis whereas the main energy source for Treg cells is oxidative phosphorylation (OXPHOS). A study found that butyrate promotes Treg cell differentiation by shifting energy metabolism from glycolysis to OXPHOS through activation of proliferator-activated receptor gamma (PPARγ) (Wen et al., 2021). The role of SCFAs is not limited to simply promoting anti-inflammatory Treg cells differentiation, but rather coordinating the immune balance of pro-inflammatory and anti-inflammatory cells. Acetate and propionate can selectively induce the differentiation of naïve CD4^+^T-cells toward Th1 cells and Th17 cells in a specific cytokine milieu. However, transfer of these SCFA-induced Th1 and Th17 cells into mice only induced mild colonic inflammation. This may be due to that acetate and propionate-induced differentiation of these effector T cells while also promoting IL-10 expression by T cells (Park et al., 2015). Therefore, although SCFAs can enhance the differentiation of pro-inflammatory T cells under certain conditions, they induce these T cells to co-express both pro-inflammatory cytokines and immunosuppressive factors, thereby preventing the colonic inflammation induced by a highly pro-inflammatory environment. In addition, the regulatory role of SCFAs also correlates with the differentiated status of CD4^+^T cells. A vitro study showed that exposing differentiated Th17 cells to butyrate can induce RORγt expression and IL-17 secretion. In contrast, exposing naïve CD4^+^T cells to butyrate in a pro-Th17 differentiation environment (IL-1β, IL-6, IL-23, TGF-β) led to downregulation of RORγt and IL-17. This may be due to the fact that naïve CD4 T cells and effector T cells have different metabolic states and express different cellular receptors on the membrane surface that can respond to environmental signals in different ways (Sałkowska et al., 2017).

#### 4.2.2 BAs

**Figure 3:**
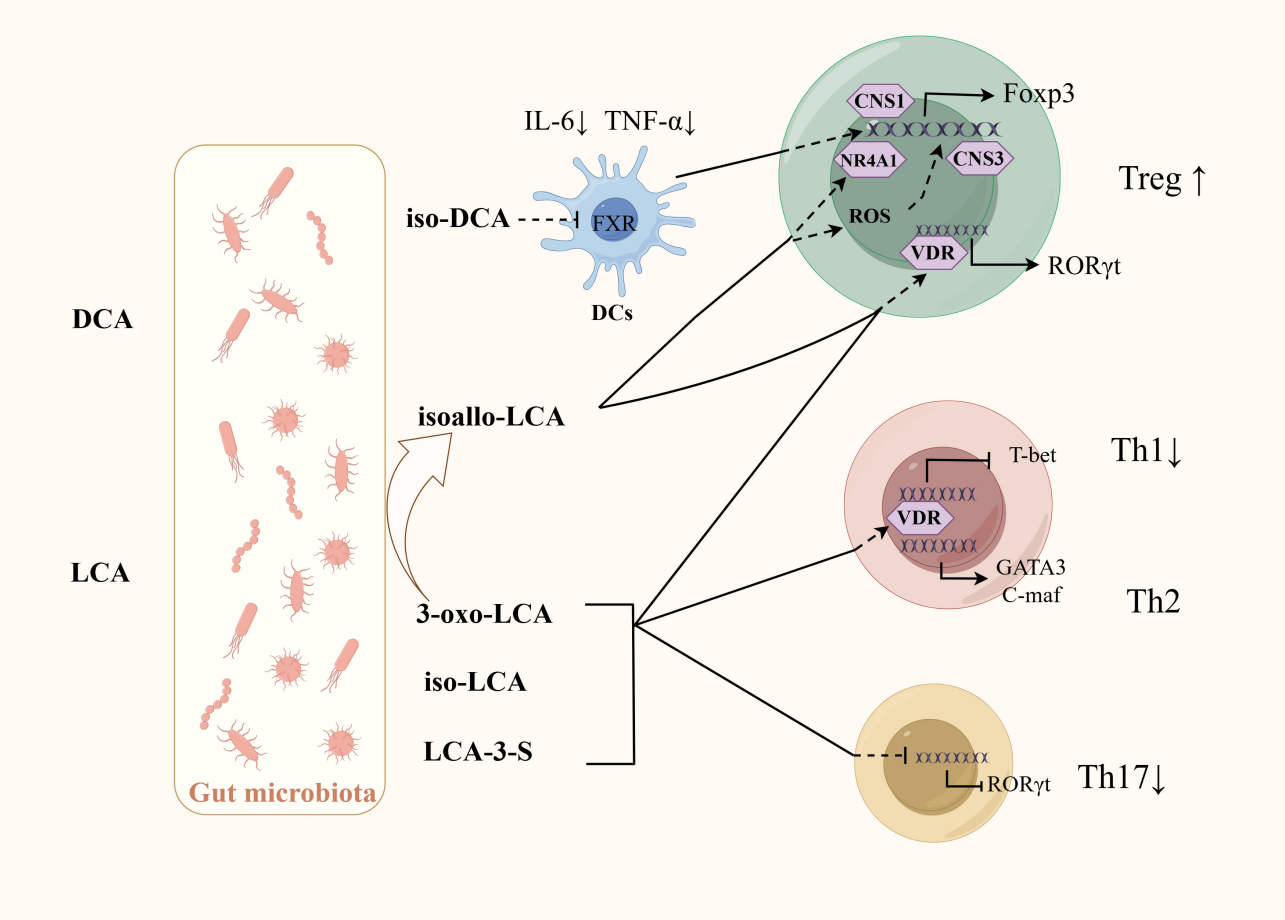
Regulation of BAs and their derivatives on the differentiation of CD4^+^T cells. GM is involved in the production of SBA and its derivatives. Iso-DCA promotes Treg cells differentiation through FXR on DCs. Isoallo-LCA promotes Treg cells differentiation through mitochondrial reactive oxygen species and NR4A1. LCA and its derivatives can inhibit Th17 cells differentiation by binding to RORγt, inhibit Th1 activation by activating VDR. VDR can also promote the transition of Th1 cells to a Th2 phenotype by increasing the production of the transcription factors GATA-3 and cMAF. Some bacteria may promote RORγt^+^Treg differentiation by activating VDR receptors through BAs.

One study has shown that using probiotics can significantly improve the composition of the GM of colitis mice, regulate BA metabolism, increasing the levels of BAs including iso-LCA, iso-CDCA, α-MCA, and hyodeoxycholic acid. A vitro experiment found that BA mixtures mediated by probiotics, rather than the microorganisms themselves, can influence Th17 cells differentiation. Administration of these BA mixtures to colitis mice can achieve similar therapeutic effects as giving probiotics, and similarly reduces Th17 cells differentiation in the colon (Yan et al., 2023).SBAs mainly include DCA and LCA, which can be further modified by GM into different derivatives, including iso-DCA, isoallo-LCA, iso-LCA, 3-oxo-LCA, etc. Iso-DCA and isoallo-LCA can promote Treg cell differentiation. Iso-DCA-mediated Treg cells production is dependent on FXR receptors on DCs (Campbell et al., 2020). Isoallo-LCA can promote Foxp3 expression by generating mitochondrial reactive oxygen species and nuclear receptor subfamily4 group A member 1 is necessary for the process (**Figure 3**) (Hang et al., 2019; Li et al., 2021c). LCA and its derivatives can inhibit the differentiation of Th17 cells. Iso-LCA and 3-oxo-LCA can bind to RORγt, preventing it from binding to the transcription start site of IL-17, thereby inhibiting the differentiation of Th17 cells (**Figure 3**) (Hang et al., 2019; Paik et al., 2022). The sulfate of LCA (LCA-3-S) exhibited better RORγt-binging ability than its oxidated metabolite (3-oxo-LCA). LCA-3-S selectively suppressed Th17 cell differentiation without influencing on Th1, Th2, and Treg cells (Xiao et al., 2022). Bacteria are involved in the transformation between different derivatives of LCA. Research found eleven genera (including *Bacillus*, *Bacteroides*, *Bifidobacterium*, *Catenibacterium*, *Collinsella*, *Eggerthella*, *Lachnospira*, *Lactobacillus*, *Parabacteroides*, *Peptoniphilus*, and *Mediterraneibacter*) belonging to the phylum Bacteroidetes are responsible for the conversion from 3-oxo-LCA to isoallo-LCA (Paik et al., 2022). LCA and its derivatives can activate the VDR, which inhibit Th1 cells activation and promote the transition of Th1 cells to a Th2 phenotype by increasing the production of the transcription factors GATA-3 and cMAF (**Figure 3**) (Boonstra et al., 2001; Pols et al., 2017). RORγt is usually associated with Th17 cells differentiation, but RORγt^+^Tregs have been observed in colonic tissues in IBD patients, where they represent a stable regulatory T-cell effector lineage with enhanced suppressive capacity during intestinal inflammation (Gomez-Bris et al., 2023). Several intestinal bacteria including *Clostridiae* and *Bacteroides* can promote the expression of RORγt^+^Treg (Sefik et al., 2015). Further studies revealed that these bacteria may promote the differentiation of RORγt^+^Treg through the activation of VDR receptors by BAs (Song et al., 2020).

#### 4.2.3 Trp

**Figure 4:**
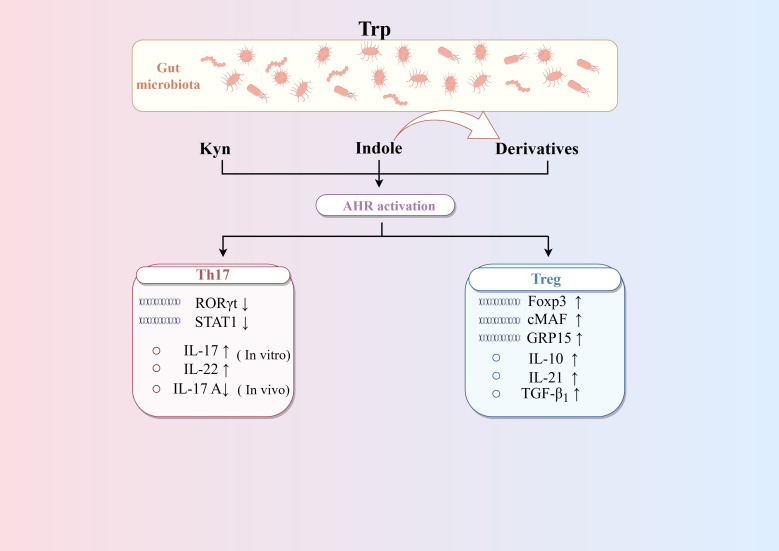
Regulation of AHR on the differentiation of CD4^+^T cells. Trp metabolites such as Kyn, kynurenic acid and indole-derived metabolites are activators of AHR. AHR can promote the differentiation of Treg cells by enhancing TGF-β secretion and Foxp3 expression, as well as Treg cells homing via GPR15. AHR can promote the differentiation of Th17 cells differentiation *in vitro* but inhibit it *in vivo*. AHR can synergize with the transcription factor cMAF to promote IL-27-mediated Tr1 cells differentiation. Activating AHR on mature Th17 cells can induce the transformation of Th17 cells into Tr1 cells by altering cytokine secretion.

AHR is the key target linking Trp metabolism and CD4^+^differentiation (**Figure 4**). Research found *Bacteroides thetaiotaomicron* increased the levels of the AHR ligands indole metabolites-indole acetic acid and indole propionic acid in DSS-induced mice, which influence the differentiation of Treg cells (Li et al., 2021b). Trp metabolites like Kyn, kynurenic acid, and indole-derived metabolites are activators of AHR. On the one hand, AHR may directly drive Treg cells differentiation by inducing Foxp3 expression (Seo and Kwon, 2023). On the other hand, AHR can promote the secreting of TGF-β_1_, which is one of the most potent inducers of Treg cells differentiation (Nguyen et al., 2010). Moreover, AHR can directly binds to open chromatin regions of the GPR15 locus to enhance its expression, which is able to mediate the homing of circulating Treg cells. In addition, AHR has also been associated with the production of Tr1 cells. AHR can synergize with the transcription factor cMAF to promote IL-27-mediated Tr1 cell differentiation, which in turn promotes IL-10 and IL-21 (Apetoh et al., 2010). However, the role of AHR in Th17 differentiation is not consistent. *In vitro* studies have shown that AHR promotes the production of IL-17 and IL-22 by Th17 cells (Veldhoen et al., 2009; Lamas et al., 2018). AHR also promotes Th17 cells differentiation by inhibiting STAT1 (Kimura et al., 2008). In contrast, an animal study argues that AHR has a role in inhibiting Th17 cells differentiation. It has been found that Th17 cells in the intestinal lamina propria are increased in the colon tissues of AHR knockout mice, suggesting that AHR plays a role in inhibiting Th17 cell differentiation *in vivo* (Qiu et al., 2013). It is worth noting that under physiological conditions, Th17 cells can undergo reprogramming and differentiate into Tr1 cells, a process that occurs during infection to help maintain homeostasis. Effector Th17 cells express high levels of AHR, the activation of which can reduce the expression of IL-17A while increasing the expression of IL-10. This shift led to the transformation of Th17 cells into Tr1 cells and helps to terminate the excessive immune response. Therefore, therapies targeting the interaction between the microbiota and AHR may help restore immune tolerance in IBD, while minimizing the harmful side effects associated with systemic immunosuppressive therapies (Gagliani et al., 2015).

### 4.3 The plasticity of GM

Interactions between GM and the CD4+T cell differentiation are extremely complex. Firstly, different GM exhibit subtle differences in inducing the differentiation of the same CD4+T cell subsets. *Candida albicans* (*C. albicans*), *Staphylococcus aureus* (*S. aureus*) (Zielinski et al., 2012), and SFB are all mucosal-associated microbiota that can induce the differentiation of Th17 cells. The Th17 cells induced by *S. aureus* and *C. albicans* show differences in cytokine secretion. Th17 cells induced by C. albicans primarily secrete IL-17 and IFN-γ but do not secrete IL-10, whereas Th17 cells induced by *S. aureus* secrete IL-17 and, upon restimulation, can transiently downregulate IL-17 levels while simultaneously secreting IL-10 (Zielinski et al., 2012). This discrepancy is related to the high IL-1β levels in the microenvironment formed by *C. albicans*.

The microbial background plays a crucial role in determining the differentiation of CD4^+^T cells. While most studies have deeply explored the mechanisms of individual bacteria, these findings may not necessarily reflect the real effects of the GM in a symbiotic context. One study used altered schaedler flora (ASF), consisting of *Bacteroides*, *Lactobacillus*, *Clostridium*, and *Mucispirillum*, to colonize into germ-free mice and observed a significant expansion and activation of Treg cells in the colonic lamina propria. However, these changes were not seen in the small intestinal lamina propria (Geuking et al., 2011). Interestingly, when ASF was colonized in mice lacking the ability to generate Treg cells, it induced the differentiation of Th17 cells. These findings suggest that the differentiation capacity of the GM is influenced by the immune status of the host and the specific segment of the gut, which may explain why the immune microenvironment in UC and CD is similar yet not identical.

The properties of the GM may explain why different strains from the same species can differentiate CD4^+^ T cells into different subtypes. *Bifidobacterium adolescentis* (*B. adolescentis)* strains IF1-11 and IF1-03 have similar adhesion capacities but different aggregation properties. The auto-aggregative *B. adolescentis* strain IF1-11 induced higher IL-6 and lower IL-10 secretion from macrophage, which increase the abundance of Th17 cells. The non-aggregating strain IF1-03 and its exopolysaccharides induced more IL-10, less IL-6 and increase the proportion of Treg cells, which is dependent on the TLR2/ERK/p38 MAPK signaling (**Figure 1**) (Yu et al., 2019). Besides, it is found that polysaccharide A (PSA) produced by *Bacteroides fragilis* can mediate the differentiation of CD4^+^T cells into different subtypes by binding to TLR receptors on various cells. PSA can bind to TLR2 on DCs to promote IL-12 secretion, which in turn drives Th1 cells differentiation, enhancing the immune response to infection. Meanwhile, PSA can bind to TLR2 on CD4^+^ T cells to directly promote Treg cells differentiation and limit excessive inflammation to maintain immune homeostasis (**Figure 1**) (Wang et al., 2006; Round and Mazmanian, 2010).

## 5 TCM ameliorates IBD through regulating GM and the differentiation CD4^+^T cells

### 5.1 Understanding IBD from a TCM theoretical perspective

Generally, IBD is categorize as “dysentery”, “abdominal pain” and “diarrhea” within the framework of TCM theory. In TCM, the pathogenesis of IBD is primarily understood as the spleen deficiency coupled with an accumulation of dampness-heat. Thus, current therapeutic approach typically emphasizes strengthening the spleen and clearing heat and dampness simultaneously in the treatment. Some studies have explored the biological underpinnings of the etiology and pathogenesis of IBD. Spleen deficiency may be associated with dysbiosis of the intestinal microbiota, while dampness-heat is thought to be linked to the activation of immune-mediated inflammation. A study found reduced abundance of *Delftia* and an increased abundance of *Lachnoclostridium* may represent key microbial signatures in UC patients with spleen deficiency, distinguishing them from individuals with other TCM patterns (Zhang et al., 2019b; Ma et al., 2020; Guo et al., 2024). Shenling Baizhu San, a classic TCM formula for the treatment of diarrhea associated with spleen deficiency, has been shown to ameliorate diarrhea in mice by modulating SCFAs through the GM (Qiao et al., 2024). In IBD patients with damp-heat symptoms, more pronounced immune-inflammatory responses are observed compared to those without damp-heat symptoms, characterized by higher levels of C-reactive protein (CRP) and erythrocyte sedimentation rate (Zhang and Shen, 2019). TCM with heat-clearing effect has been found to reduce a range of pro-inflammatory cytokines in TNBS-induced colitis in mice (Hu et al., 2024). These findings suggest that the association between GM dysregulation and immune inflammation is the biological basis of the TCM pathogenesis of IBD, highlighting the potential therapeutic value of TCM in treating IBD by modulating the immune response mediated by GM.

### 5.2 TCM improves inflammation in IBD patients

Substantial clinical trials have supported the effectiveness of TCM in managing IBD. A systematic review included 28 randomized controlled trials of 18 herbal ingredients and found that some herbs with translational potential, such as turmeric, indigo, liquorice, and pomegranate peel (Iyengar et al., 2024). An open-label study enrolled 11 patients with refractory UC who were treated with oral indigo, of whom 10 achieved clinical remission and all patients showed endoscopic improvement (Saiki et al., 2021). Qingchang suppositories, consisting of *indigo*, *panax notoginseng*, *purslane* et al, have demonstrated superior clinical efficacy in treating UC compared to salicylazosulfapyridine suppositories, significantly improving symptoms and CRP levels, as well as promoting mucosal healing (Shen et al., 2021; Xiaoling et al., 2024). Another study reported that the efficacy of TCM in treating UC may be related to CD4 cells. Qingchang Xiaopi Decoction was reported to be capable of effectively relieving diarrhea and abdominal pain in patients with mild to moderate UC and reducing the levels of Th17 cell-related cytokines IL-6, IL-17, and IL-23 in serum (Jia et al., 2024). However, most current research on TCM focus on UC, their clinical efficacy of CD still needs further evaluation (Zeng et al., 2022).

### 5.3 TCM improve intestinal inflammation through modulating GM profile and CD4^+^ T cells

Many studies have demonstrated that Chinese herbal extracts and TCM formulas can help may rescue patients from intestinal immune inflammation by modulating CD4^+^T cells through the GM and its metabolites (**Table 2**). Despite the immunological and GM differences between humans and animals, DSS and TNBS remain the most commonly used models for studying IBD. These models can satisfactorily mimic the overactive immune response, intestinal barrier disruption, and GM dysbiosis characteristics observed in IBD (Kolios, 2016). Similar to the microbial changes in humans with IBD, a decrease in beneficial bacteria including Bacteroidetes, Clostridium, and Lactobacillus can be observed in these models (De Fazio et al., 2014). Bawei Xileisan can regulate the Treg/Th17 balance in a GM-dependent manner. The increased abundance of Treg cells and decreased abundance of Th17 cells by Bawei Xileisan may be may be associated with the restoration of *Lactobacillus* and *Bacteroides* (Wen et al., 2016). Gegen Qinlian decoction (GQD) can restore the Treg/Th17 and Th2/Th1 balance and reduce related cytokines in TNBS-induced colitis. The immunomodulatory effects of GQD may be achieved by the suppressed overgrowth of pathogenic bacteria, such as *Helicobacter*, *Proteobacteria*, and *Mucispirillum*, and improved abundance of beneficial bacteria, including *Lactobacillus*, *Muribaculaceae*, *Ruminiclostridium*, *Akkermansia*, and *Ruminococcaceae* (Hu et al., 2024). Furthermore, TCM has been shown to inhibit the interaction between Th1 and Th17 cells. Wumei Decoction (WMD) can inhibit the synergistic effects of Th1 and Th17 cells by increasing the abundance of *Allobaculum* and *Bacteroides*, which effectively terminated DSS-induced colitis (Wu et al., 2022).

**Table 2:** The mechanism of TCM extracts and TCM formula.

TCM formula or extracts	Main components	Model	Alterations in gut microbiota	Alterations in CD4^+^T cell	Alterations in cytokines	Reference
Wumei decoction	*Mume Fructus*, *Asari Radix et Rhizoma*, *Zingiberis Rhizoma*, *Coptidis Rhizoma*, *Angelicae Sinensis Radix*, *Typhonii Rhizoma*, *Zanthoxyli Pericarpium*, *Ramulus Cinnamomi*, *Phellodendri Chinensis Cortex*, *Ginseng Radix et Rhizoma*	DSS mice	Genus: *Allobaculum, Bacteroides*↑ *Ileibacterium* ↓	Th1, Th17 ↓	IL-17A, TNF-α, IFN-γ, IL-1β↓	(Wu et al., 2022)
Kuijie decoction	*Radix Cynanchi Paniculati*, *Herba Portulacae*, *Radix Caraganae*, *Radix Sanguisorbae*, *Flos Sophorae*, *Salvia Miltirrhizae*, *Setariae Fructus Germinatus*	DSS mice	Family: Lachnospiraceae ↑ Genus: *Lachnospiraceae* *- NK4A136 group*↑ Trp↑	Treg↑ Th17↓	IFN-γ, TNF-α, IL-1β, IL-6 ↓	(Peng et al., 2024)
Rhubarb Peony Decoction	*Rhei Radix et Rhizoma*, *Moutan cortex*, *Persicae semen*, *Natrii sulfas*, *Benincasae semen*	DSS mice	Phylum: Firmicutes, Actinobacteria↑ Proteobacteria, Bacteroidetes ↓ Species: *Butyricicoccus, pullicaecorum*↑	Treg↑ Th17↓	IL-6, TNF-α, IFN-γ, IL-10, IL-17A, IL-21, IL-22↓ TGF-β↑	(Luo et al., 2019)
Gegen Qinlian Decoction	*Pueraria lobata*, *Scutellaria baicalensis*, *Coptis chinensis*, *Glycyrrhiza uralensis*	TNBS mice	Phylum: Firmicutes, Verrucomicrobia↑ Bacteroidetes, Proteobacteria, Deferribacteres↓ Genus: *Lactobacillus, Muribaculaceae, Ruminiclostridium, Akkermansia* ↑ *Helicobacter, Proteobacteria*, *Mucispirillum↓*	Th2, Th1, Th17↓ Treg ↑	IL-10 ↑ IL-17A, IL-2, IL-5, IL-6, IL-13↓	(Hu et al., 2024)
Modified Gegen Qinlian Decoction	*Pueraria lobata*, *Scutellaria baicalensis*, *Coptis chinensis*, *Glycyrrhiza uralensis*, *Zingiberis Rhizoma*, Talcum	DSS mice	Family: Ruminococcaceae↑ Genus: *Lactobacillus* ↑ *Bacteroides* ↓ Species: *Escherichia-Shigella, Clostridium_Sensu_Stricto_1 ↓* *Lachnospiraceae_NK4A136_group*↑ Acetate, propionate, butyrate, isobutyrate, isovalerate↑	Treg↑ Th17↓	TGF-β, IL-4 ↑ IL-17A, IL-21 ↓	(Wang et al., 2021)
Yiyi Fuzi Baijiang formula	*Coix seed*, *Radix Aconiti Lateralis*, *Patrinia villosa*	TNBS rat	Cholic acid, Taurocholic acid *↓* Glycocholic acid ↑	Th17 ↓	IL-17A, IL-21, IL-22, IL-6, TNF-α, IL-1β, IL-18 ↓	(Liu et al., 2023b)
Bawei Xileisan	Watermelon frost, calcite, cow gallstone, pearl powder, borax, *Indigo naturalis*, borneol	DSS mice	Genus: *Lactobacillus*, *Bacteroides* ↑	Treg↑ Th17↓	IL-17A, IL-17F, IL-22 ↓	(Wen et al., 2016)
Zuojin Pill	*Coptis chinensis*, *Evodia rutaecarpa*	DSS mice	Phylum: Actinobacteria ↑ Family: Verrucomicrobiaceae, Desulfovibrionaceae ↑ Class: Betaproteobacteria, Sphingobacteriia↑ Genus: *Akkermansia* ↑	Treg ↑	IL-2, IL-6, IL-17A, IL-4 ↓	(Zhou et al., 2020)
Xuanfei Baidu Decoction	*Ephedrae Herba*, *Polygoni Cuspidati Rhizoma et Radix*, *Glycyrrhizae Radix et Rhizoma*, *Coicis Semen*, *Gypsum Fibrosum*, *Atractylodis Rhizoma*, *Artemisia Annua Herba*, *Pogostemonis Herba*, *Descurainiae Semen Lepidii Semen*, *Verbenae Herba*, *Phragmitis Rhizoma, Exocarpium*, *Armeniacae Semen Amarum*	DSS mice	Family: Lachnospiraceae, Muribaculaceae↑ Genus: *Akkermansia*, *Enterorhabdus*↑ *Turicibacter* ↓ Species: *Escherichia-Shigella*, *Eubacterium nodatum, Clostridium sensu stricto 1*↓	Th1↓ Th2↑	TNF-α↓	(Ma et al., 2022)
Xianglian pill	*Coptidis Rhizoma*, *Evodia Rutaecarpa*, *Radix Aucklandiae*	DSS rat	Phylum: Bacteroidetes, Verrucomicrobia ↑ Firmicutes ↓ Genus: *Bacteroides, Phascolarctobacterium* ↑	Treg ↑	TNF-α, IL-6 ↓ IL-10 ↑	(Liu et al., 2023a)
Stigmasterol	/	DSS mice	Genus: *Ruminococcus*, *Prevotella*, *Paraprevotella*, *Helicobacter*, *Odoribacter* ↑ *Streptococcus*, *Escherichia*, *Enterococcus, Allobaculum* ↓ Species: *Clostridium*_IV and XlVa ↑ Acetate, propionate, butyrate, isobutyrate, valerate↑	Treg ↑ Th17↓	IL-10, TGF-β↑ IL-17A ↓	(Wen et al., 2021)
Liquiritin apioside	/	DSS mice	Phylum: Firmicutes↑ Bacteroidete ↓ Family: Lachnospiraceae, Erysipelotrichaceae, Muribaculaceae, Lactobacillaceae, Marinifilaceae ↑ Bacteroidaceae ↓ Genus: *Lachnospiraceae NK4A136 group, norank_f:Muribaculaceae, Turicibacter*, *Lactobacillus, Odoribacter*↑ Acetate, butyrate and isobutyrate ↑	Treg↑ Th17↓	IL-1β, IL-6, TNF-α, IL17A↓ IL-10↑	(Xia et al., 2023)
Abelmoschus manihot	/	DSS mice	Phylum: Firmicutes↑ Bacteroidetes↓ Genus: *Bacteroides*, *Alistipes*, *Lactobacillus*, *Bilophila*, *Desulfovibri* ↑ Acetate, butyrate↑	Treg↑ Th17↓	IL-17, IL-22, IL-23↓ IL-10, TGF-β↑	(Zhang et al., 2019a)
Baicalin	/	TNBS rat	Phylum: Firmicutes↑ Proteobacteria Actinobacteria↓ Genus: *Butyricimonas*, *Roseburia*, *Subdoligranulum*, *Eubacterium*↑ Acetate, propionate, butyrate↑	Treg↑ Th17↓	IL-10 ↑ IL-17 ↓	(Zhu et al., 2020)
Salidroside	/	DSS mice	Phylum: Firmicutes↑ Bacteroidetes↓ Family: Lachnospiraceae Ruminococcaceae↑	Treg↑	IL-1β, IL-17A, IL-6, TNF-α, IFN-γ↓ IL-10↑	(Liu et al., 2023c)
Astragalus polysaccharides	/	DSS mice	Family: Muribaculaceae, Lachnospiraceae, Rikenellaceae, Ruminococcaceae ↑ Genus: *Prevotellaceae_UCG-001*, *Alistipes, Rikenellaceae_RC9_gut_group*, *Muribaculum* ↑ *Bacteroides* ↓ Acetate, propionate, n- butyrate, isobutyrate, valeric acid ↑	Treg↑ Th17↓	IL-1β,IL-17A, IL-6,TNF-α, IL-23↓ IL-10↑	(Zhang et al., 2025)
Berberine	/	DSS mice	Genus: *Desulfovibrio* ↓ *Eubacterium*, *Bacteroides* ↑	Treg↑ Th17↓	IL-17↓ IL-10↑	(Cui et al., 2018)
Resveratrol	/	TNBS mice	Genus: *Ruminococcus*c Species: *Ruminococcus gnavus*, *Akkermansia muciphila* ↑ *Bacteroides acidifaciens* ↓ Acetate and i-butyrate↑	Treg↑ Th17↓	IFN-γ, IL-17↓ IL-10↑	(Alrafas et al., 2019)

↑, upregulated; ↓, dwonregulated.

**Figure 5:**
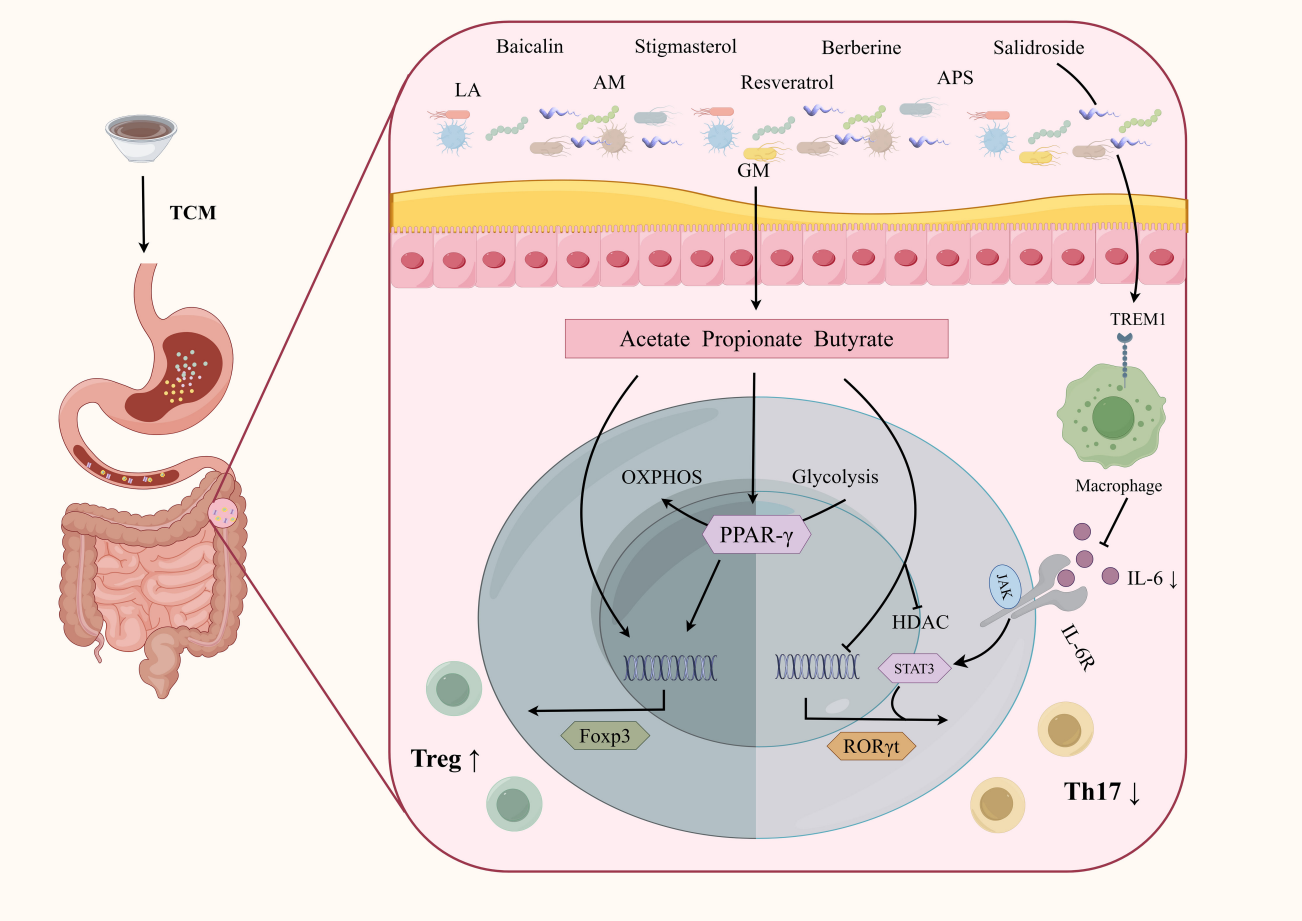
TCM interferes CD4^+^T cells differentiation by regulating GM in the treatment of IBD. TCM can affect CD4^+^T cell differentiation by influencing GM and their metabolites, which mainly include the promotion of Treg cells and inhibition of Th17 cells. Although further studies are needed, targeting CD4^+^T cells through the GM represents a promising therapeutic direction for TCM in the treatment of IBD. APS, Astragalus polysaccharides; LA, Liquiritin apioside; AM, Abelmoschus Manihot.

TCM can also regulate CD4^+^ T cell differentiation by modulating GM metabolites (**Figure 5**). Stigmasterol is a plant-derived sterol extracted from Chinese herbs such as *Scutellaria baicalensis* Georgi and *Phellodendron chinense* Cortex (Bakrim et al., 2022). Stigmasterol can promote the production of SCFA, especially butyrate, by regulating GM (Wen et al., 2021). *Abelmoschus manihot* can also restore the Th17/Treg balance by increasing the level of SCFA-producing bacteria such as *Lachnospiraceae* (Zhang et al., 2019a). Liquiritin apioside (LA) is a flavonoid component extracted from licorice (Yang et al., 2017). Xia et al. demonstrated that LA administration altered the composition of GM at the phylum, family, and genus levels. The increased abundance of *Muribaculaceae*, *Lachnospiraceae* NK4A136 group, *Odoribacter*, and *Lactobacillus* promoted SCFAs production, thereby modulating the Treg/Th17 balance in DSS-induced colitis (Xia et al., 2023). Modified GQD significantly increased the abundance of SCFA-producing GM, promoted Treg cells differentiation, and inhibited Th17 cells differentiation. Notably, this protective effect was not observed in mice that were depleted of GM using a broad-spectrum antibiotic mixture, further highlighting the critical role of SCFA-producing microbiota (Wang et al., 2021). Rhubarb Peony Decoction has also been reported to restore the Th17/Treg balance and regulate GM dysbiosis, specifically by enhancing the abundance of the butyrate-producing species *Butyricicoccus pullicaecorum* (Luo et al., 2019). Kuijie Decoction, a prescription composed of eight Chinese herbs, has been shown to restore Th17/Treg homeostasis, regulate GM dysbiosis, and increase the levels of metabolites such as glutamine and Trp (Peng et al., 2024).

### 5.4 Specific molecular mechanism of TCM components in regulating microbiome-immune axis

Previous studies have shown that TCM prescriptions and extracts have effects on GM and CD4. We are more focused on the molecular mechanism of TCM to explore reliable druggable targets. One study explored tissue distribution of the main active ingredients of GQD in mice after oral administration and found the contents of baicalin, puerarin, berberine, and glycyrrhizic acid were high in the colon, which can be regarded as the main active ingredients of GQD for the treatment of intestinal disease (Lu et al., 2022). In another study, the anti-inflammatory active ingredients of GQD were screened by high-throughput in the zebrafish model of inflammatory bowel disease. Among the 7 active ingredients identified, baicalin, puerarin, berberine, and glycyrrhizic acid were further found to significantly reduce the expression of interleukins and chemokines in the colon (Yu et al., 2021). Another study applied multi-omics sequencing and found that GQD and baicalin had similar effects on intestinal flora and microbiome. Interestingly, transcriptomics results showed that the differentially expressed genes caused by GQD and baicalin were both enriched in T cell activation, indicating the baicalin as the main active ingredient of GQD in modulating microbiome-immune axis (Xu et al., 2020).

Baicalin, derived from Scutellaria baicalensis Georgi, has been shown to attenuate the TNBS-induced colitis, accompanied by downregulation of the Th17/Treg ratio, increased SCFAs levels, and improved GM dysbiosis. Specifically, butyrate-producing bacteria such as *Butyricimonas, Roseburia, Subdoligranulum*, and *Eubacterium* at the genus level were significantly enriched following baicalin administration (**Figure 5**) (Zhu et al., 2020). Resveratrol treatment could reverse the increase in the number of *Bacteroides acidulans* and the decrease in the number of *Ruminococcus gnavus* and *Akkermansia mucinphilia* caused by TNBS administration, and increase the production of isobutyric acid. Subsequent fecal transfer experiments confirmed that the resveratrol-induced microbiota prompted recipient mice to show polarization of CD4^+^FOXP3^+^T cells in response to TNBS, as well as a decrease in CD4^+^IFN^+^ and CD4^+^IL-17^+^T cells (**Figure 5**) (Alrafas et al., 2019).

## 6 Discussion

IBD is a disease that results from the interaction of genetic susceptibility and environmental factors, leading to an imbalance in immune homeostasis. Our review found that regulating the immune response via GM modulation of CD4+T cells holds significant promise, as it allows for a multi-pronged approach to restore immune balance such as stimulating APCs to secrete anti-inflammatory cytokines, and regulating key transcription factors through metabolites. In recent years, TCM has shown remarkable potential and unique advantages in regulating immune response balance. We offer a new perspective on the immunomodulatory role of TCM based on the GM-immune axis. Modern studies, based on the perspectives of GM and immune inflammation, have explored the connotation of the TCM pattern of spleen deficiency and dampness-heat in IBD. Further pharmacological research has revealed that TCM regulates GM across multiple taxonomic levels, influencing the level of related cytokines and metabolites, which in turn modulate CD4+T cell differentiation, activation, and alleviate intestinal inflammation.

These findings have profound implications for clinical practice. Firstly, certain GM and metabolites show important regulatory effects, making them potential candidates for targeted drug development. Secondly, in traditional IBD treatment, immunosuppressive drugs are often used to control the overactivation of CD4^+^T cells. While these drugs effectively manage symptoms, long-term use can lead to immunosuppression and increased risk of infections. TCM, by finely coordinating the interaction between the GM and the immune system, may serve as a complementary therapy to reduce the reliance on traditional drugs. Finally, some active extracts from Chinese herbs have demonstrated significant therapeutic effects, which can be considered as priority treatment options in clinical practice and provide new ideas for drug development.

However, despite the unique potential of TCM in regulating CD4^+^T cell immune responses, there are still challenges in its practical application. Firstly, TCM formulas contain various bioactive compounds, which complicates the study of their mechanisms. Current research has not yet fully elucidated how these compounds interact with immune pathways and how they cooperate. Second, previous studies have shown significant heterogeneity in the microbiota characteristics of IBD patients, which increases the difficulty in effectively applying TCM. Future research should focus on exploring the molecular basis of individual differences and TCM patterns, and identify key targets for TCM intervention to lay the foundation for precision medicine. Another import concern is the safety of TCM components. Most studies reported that the incidence of side effects was similar between the treatment and control groups. However, in a study that involved 877 UC patients treated with Indigo naturalis, 40 patients reported hepatic impairment, and 11 reported pulmonary artery hypertension. Although these adverse effects were reversible after discontinuing Indigo naturalis, they indicate that it is equally important to monitor the safety of herbal components (Naganuma et al., 2018). Thus, strict toxicological studies and multi-center, large-scale clinical trials are needed to assess potential safety risks. Finally, there has been less attention on TCM’s role in treating CD. Future research could explore the mechanisms of TCM in treating CD.

In conclusion, we comprehensively summarize the relationship between GM and CD4^+^T cell-mediated inflammation in IBD, as well as the regulatory role of TCM in this process. The aim is to provide a solid theoretical foundation and innovative ideas for IBD mechanism research and drug development, paving the way for more precise and effective treatment options.
